# A Mixed Osseous-Fibrous Talocalcaneal Coalition With a Posterior Extra-articular Bridge in a Nine-Year-Old: Resection via a Posteromedial Approach Augmented With Fat Interposition

**DOI:** 10.7759/cureus.98940

**Published:** 2025-12-10

**Authors:** Patar P Oppusunggu, Gabriel K Wienanda, Karina S Gani, Mitchel Mitchel, Erica Kholinne

**Affiliations:** 1 Orthopaedics and Traumatology, Gatam Institute, Eka Hospital, Tangerang, IDN; 2 Orthopaedics and Traumatology, Eka Hospital Cibubur, Bogor, IDN; 3 Faculty of Medicine, Universitas Trisakti, Jakarta, IDN

**Keywords:** fibrous, osseous, pediatric, talocalcaneal coalition, tarsal coalition

## Abstract

Tarsal coalition is a congenital condition and is an often underrecognized cause of pediatric foot pain, most frequently involving the calcaneonavicular and talocalcaneal joints, while mixed-type coalitions are exceptionally uncommon and are rarely reported.

We present the case of a nine-year-old boy with progressive left foot pain and limping, unresponsive to conservative treatment. Imaging confirmed a mixed-type talocalcaneal coalition with both bony and fibrous components. Surgical management consisted of osteotomy, coalition resection, and fat interposition via a posteromedial approach, chosen for its direct access to the posterior and medial facets while minimizing tendon and neurovascular risk. Preoperatively, the patient demonstrated hindfoot valgus of 13.28°, restricted subtalar motion, and Oxford Ankle Foot Questionnaire for Children (OxAFQ-C) scores of 12.5% (physical), 56.3% (school/play), and 55% (emotional). At four weeks postoperatively, subtalar mobility had improved to increased eversion and inversion, hindfoot alignment was corrected, and the patient was scheduled for muscle strengthening, functional retraining, and dynamic weight-bearing physiotherapy. By four months, OxAFQ-C scores had risen to 83.3%, 87.5%, and 87.5%, respectively, reflecting significant functional recovery and enhanced quality of life.

## Introduction

Tarsal coalition is an abnormal connection between two or more tarsal bones, which may be osseous, cartilaginous, or fibrous. It is often an underrecognized cause of foot and ankle pain [1]. The most common types of tarsal coalition are calcaneonavicular (53%) and talocalcaneal (37%), together accounting for 90% of all cases [2]. In about 50% of cases, a tarsal coalition occurs bilaterally [3]. Tarsal coalition is a rare condition, affecting less than 1% of the population, and may be associated with other congenital malformations. However, the true prevalence is uncertain, as most cases remain asymptomatic [4,5]. Only a few studies have described mixed-type coalitions occurring in the same foot. A retrospective study reported that mixed-type talocalcaneal coalitions account for only 5% of all cases [6]. Tarsal coalition should be included in the differential diagnosis for children presenting with flatfoot or recurrent ankle sprains. Modern diagnostic evaluation primarily depends on advanced imaging techniques, with computed tomography (CT) considered the gold standard. The condition usually becomes evident in children who show restricted subtalar motion and a submalleolar prominence, most commonly during preadolescence, when tarsal bone ossification progresses. Once symptoms develop, surgical intervention is generally recommended to prevent further complications.

In this case report, we present a rare instance of a nine-year-old boy with left symptomatic mixed-type talocalcaneal coalition, without any associated congenital anomalies or syndromic conditions, who failed conservative treatment with an insole, physiotherapy, and analgesics for six months, and was released with an operative approach with fat interposition.

## Case presentation

A nine-year-old boy presented to the orthopedic outpatient clinic with a two-month history of left foot pain and limping during walking, which had progressively worsened over the past two weeks. The pain was exacerbated by standing or walking on tiptoes, and there was no tingling sensation or numbness in the foot. He had previously undergone physiotherapy and received conservative treatment, but these interventions yielded no significant improvement.

On physical examination, a unilateral antalgic gait was observed in this patient, who exhibited pes planus with 13.28° of hindfoot valgus alignment. Localized tenderness was noted over the medial aspect of the left talocalcaneal joint, accompanied by restricted subtalar range of motion (ROM) and pain elicited during active and passive inversion and eversion. Passive testing of the talocalcaneal joint revealed no appreciable movement. The "too many toes" sign was also observed in this patient, with the absence of peroneal spasm. We administered the Oxford Ankle Foot Questionnaire for Children (OxAFQ-C) preoperatively to assess surgical outcomes [7]. This questionnaire consists of three domains: physical, school and play, and emotional. The patient’s preoperative scores were 12.5% in the physical domain, 56.3% in the school and play domain, and 55% in the emotional domain.

**Figure 1:**
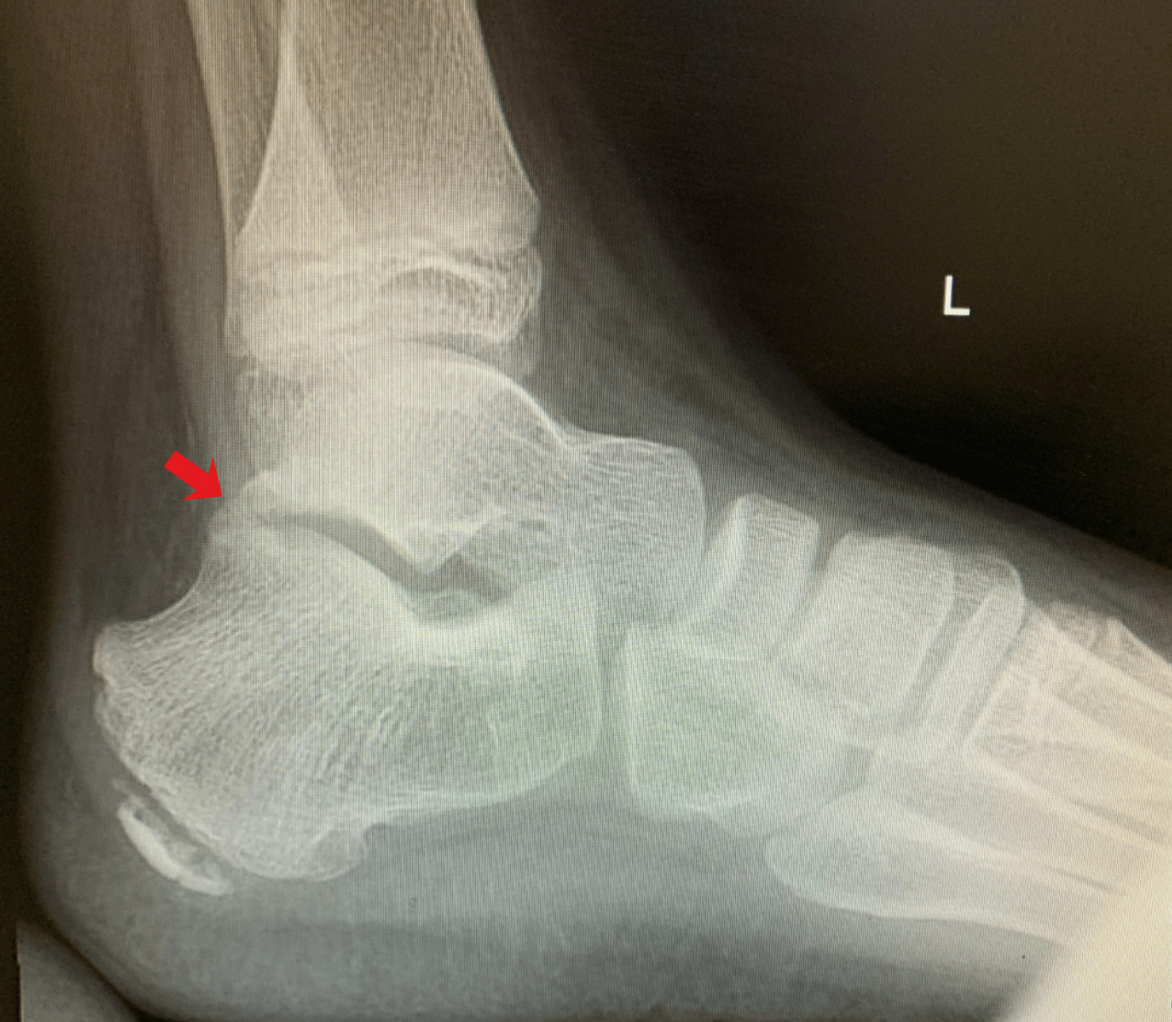
Weight-bearing lateral view of the left ankle demonstrating posterior bony bridging (red arrow).

**Figure 2:**
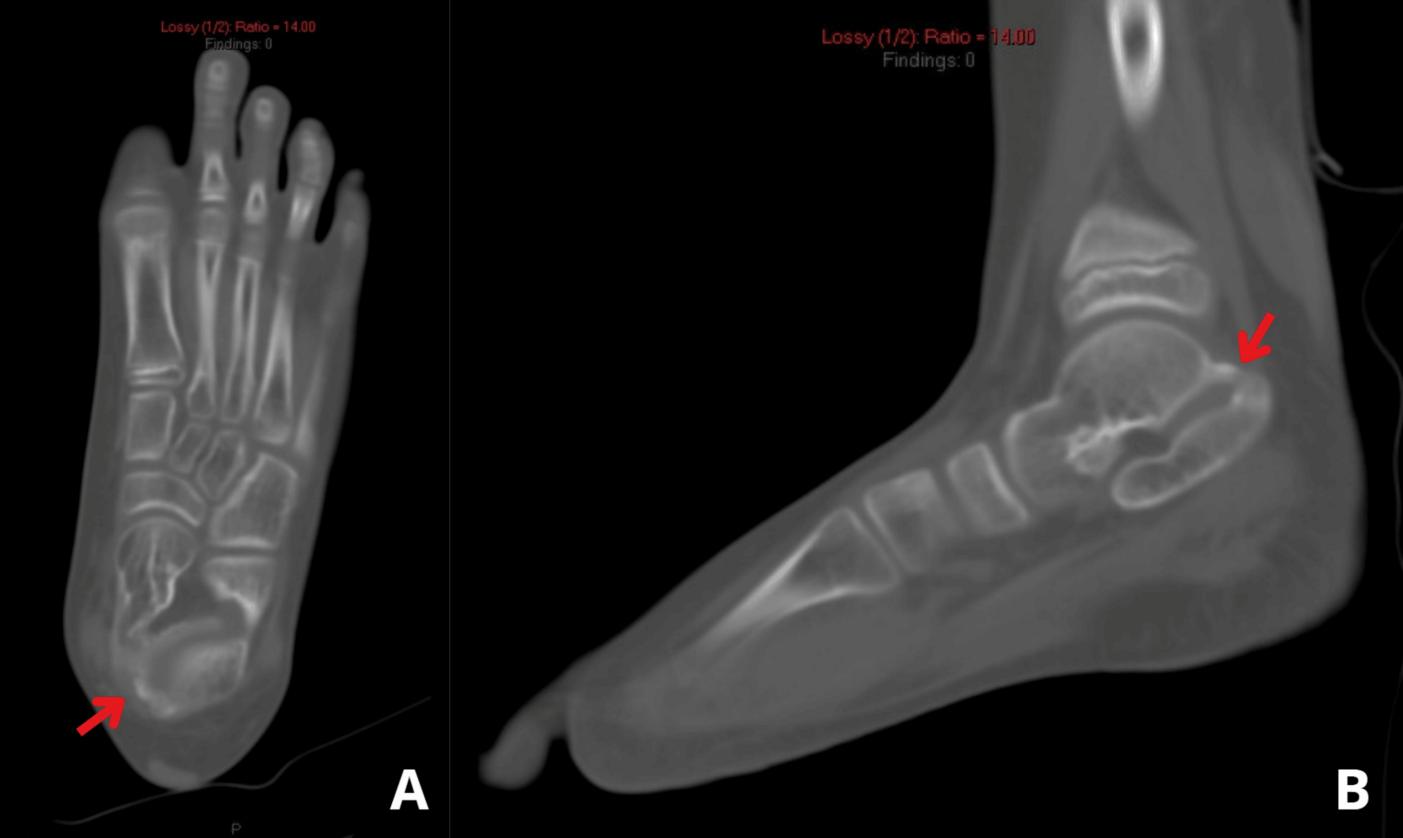
Computed tomography of the left foot demonstrating (A) a transverse view and (B) a sagittal view, revealing an osseous talocalcaneal coalition (red arrow).

**Figure 3:**
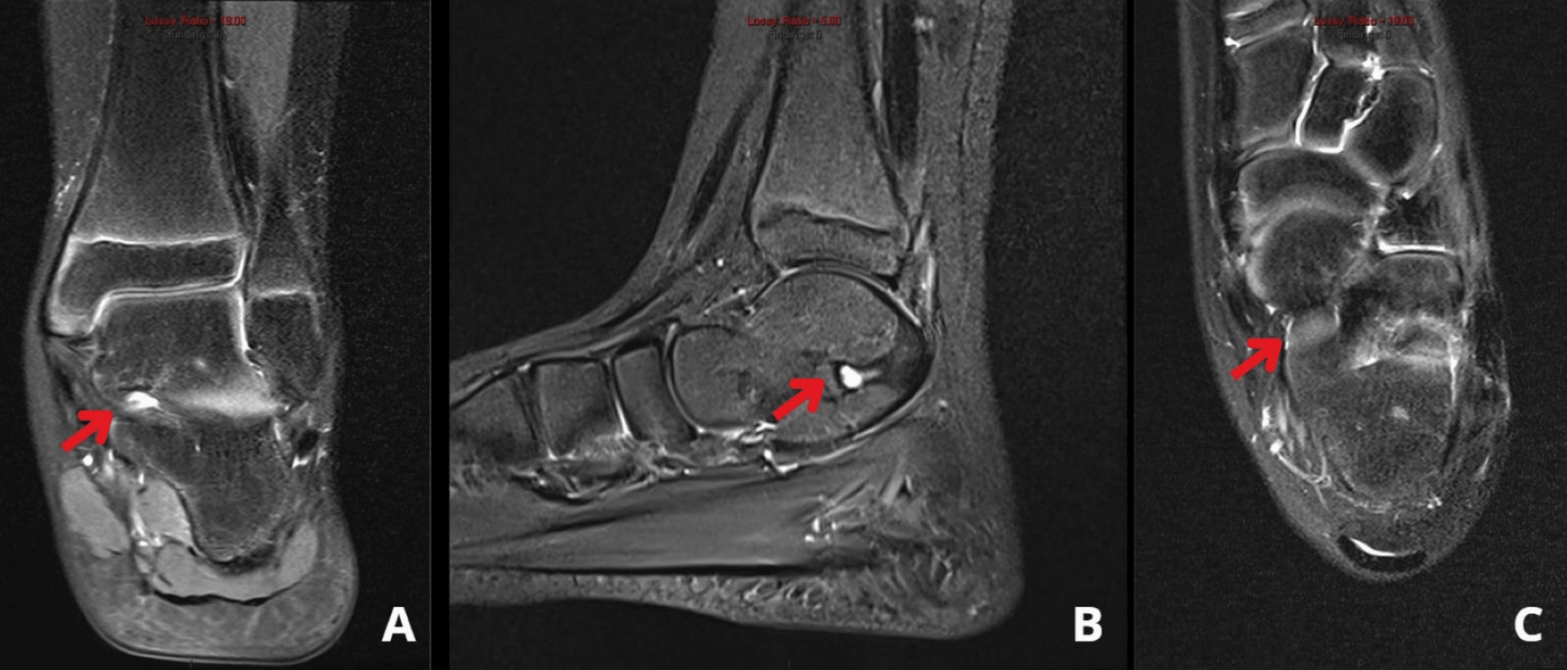
Magnetic resonance imaging of the left foot demonstrating (A) a coronal view, (B) a sagittal view, and (C) a transverse view, revealing a fibrous talocalcaneal coalition (red arrow).

Plain radiographs demonstrated a posterior bony bridging in the talocalcaneal joint (Figure 1). CT and magnetic resonance imaging (MRI) revealed a bony coalition involving the whole posterior extra-articular facet, as well as fibrous coalitions involving 60% of the medial side of the talocalcaneal joint (Figures 2-3).

Surgical technique

An operative procedure was scheduled to perform osteotomy and resection of the talocalcaneal coalition involving both bony and fibrous components on the left foot. Under general anesthesia, the patient was positioned supine with standard sterile draping. A pneumatic tourniquet was applied proximally and inflated to 200 mmHg for bleeding control.

**Figure 4:**
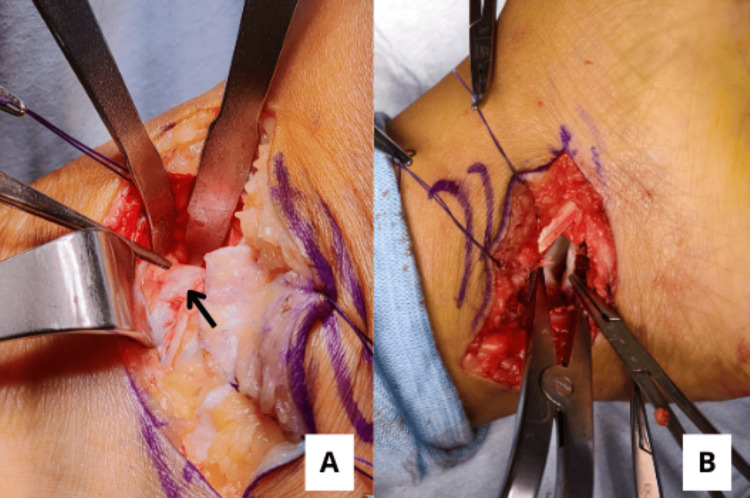
Intraoperative images showing (A) a fibrous coalition (black arrow) inside the talocalcaneal joint, and (B) complete release of both fibrous components within the talocalcaneal joint following osteotomy and resection.

A posteromedial incision was made approximately 2 cm posterior to the medial malleolus, based on the location of the bony and fibrous coalition. The flexor digitorum longus is retracted either dorsally or plantarly, depending on the coalition’s location and ease of exposure. The flexor hallucis longus (FHL) is retracted plantarly, away from the sustentaculum tali. The posterior margin of the middle facet is then bluntly identified, and a retractor is positioned to define the posterior boundary of the pathologic facet, while also protecting the FHL and posterior tibial neurovascular bundle. Another retractor is placed at the anterior edge of the middle facet to isolate and expose the coalition fully. Intraoperatively, a bony bridge was identified at the posterior aspect of the joint, while a fibrous coalition was observed medially (Figure 4A). Osteotomy and resection were performed from the anteromedial to the posteromedial aspect of the joint to release both fibrous and bony components of the coalition. The resection was carried out until a clear gap was observed across the resection site (Figure 4B).

**Figure 5:**
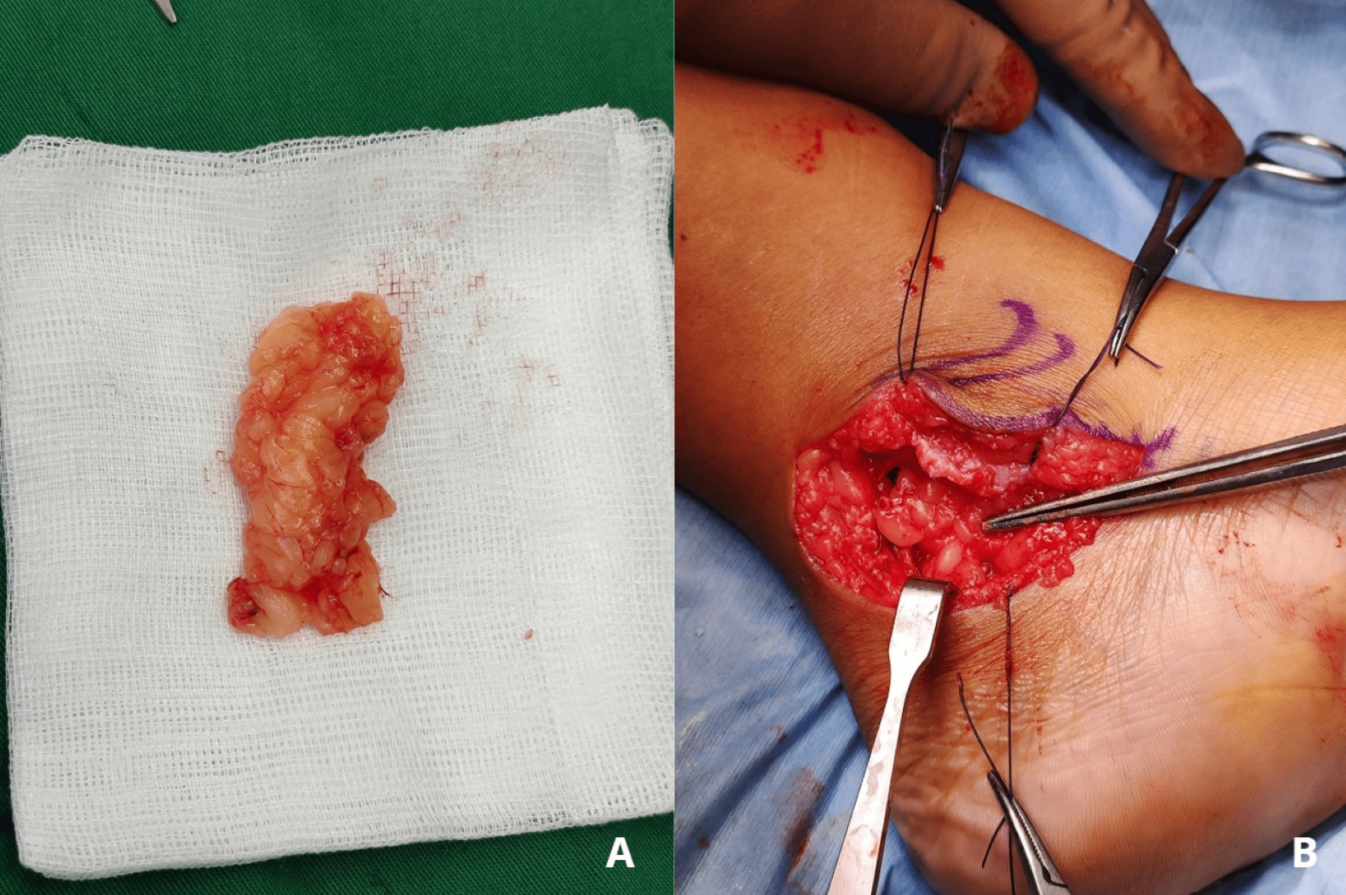
(A) Fat graft harvested from the posterior calf. (B) Fat graft implanted into the talocalcaneal joint and secured to the surrounding tissue.

Passive intraoperative assessment of joint mobility, following the resection, revealed restoration of the normal ROM. Importantly, the articular surfaces were preserved, with no evidence of iatrogenic damage. A fat graft measuring 5 × 3 × 0.7 cm (length × width × thickness), harvested from the posterior calf for its aesthetic benefit and rich fat content, was implanted into the resected talocalcaneal joint (Figure 5). The graft was secured in place with capsular sutures, and its position between the talus and calcaneus was visually confirmed throughout arcs of motion to ensure proper interposition. The incision was then closed in layers, using standard suturing techniques. The patient was immobilized using a backslap and cast for four weeks. We have summarized a key surgical step and potential pitfalls in talocalcaneal coalition resection in Table 1.

**Table 1:** Key surgical steps and potential pitfalls in talocalcaneal coalition resection.

Step	Key Steps	Pitfalls
Identification & Mobilization of Tibial Nerve/Posterior Tibial Vessels, Flexor Digitorum Longus (FDL), Flexor Hallucis Longus (FHL)	Assess the posterior aspect of the medial malleolus, then perform blunt dissection to identify the tendon located just beneath it [8]. Extend the lesser toes and great toe to identify the FDL and FHL by observing tendon movement within their sheaths, then gently retract them to expose and assess the surrounding neurovascular structures within the fatty tissue [9]. The tibial neurovascular bundle is identified and gently retracted [9].	Misidentifying or not identifying the FDL and FHL risks tendon injury. Excessive traction on the tibial neurovascular bundle may cause neuropraxia, ischemia, or pain. Additionally, incomplete tendon mobilization limits exposure and increases iatrogenic injury risk, while inadequate protection of the neurovascular bundle can result in laceration or compression [10].
Resection Corridor (Anteromedial → Posteromedial) with Cartilage Preservation	Establish a corridor across the coalition from anteromedial to posteromedial [10]. Use a C-arm with a small Abbocath to confirm the joint space and assess the margins of the calcaneus and talus [8,11]. Use small rongeurs until a clear gap is achieved [8]. Preserve all the joint structures [8].	Over-resection may result in articular cartilage injury, leading to early-onset arthritis and potential ankylosis [8]. Poor visualization may lead to incomplete resection, resulting in recurrence and chronic pain syndrome [8].
Landmarks for Extent of Resection	The coalition is carefully released in a stepwise manner from superficial to deep using a fine bone curette until the articular surface is clearly visualized. After creating the cleft window, a spacer or distractor is inserted under fluoroscopic (C-arm) guidance to maintain separation and minimize the risk of articular surface injury [12].	Inadequate resection may result in residual coalition and persistent symptoms, whereas excessive resection can compromise structural support and lead to subtalar instability [12].
Hemostasis & Prevention of Graft Migration	The graft is secured by suturing its edges to the surrounding periarticular soft tissues, after which joint stability is evaluated using provocative maneuvers of the talocalcaneal joint, ensuring that all tendons glide smoothly and the nerves are free of entrapment [8].	Insufficient hemostasis may lead to hematoma formation, subsequent fibrosis, and potential recurrence, while inadequate graft containment can result in graft migration, compromise the tendon glide, neurovascular compression, and diminished interpositional effectiveness [8,12].

Postoperatively, the patient reported mild pain with a visual analog scale (VAS) score of 3/10 while using a back slab and remaining non-weight bearing. The pain was aggravated by activity and joint motion but subsided with rest and oral analgesic medication. At four weeks postoperative, the back slab and cast were removed, and the patient was referred to the physiotherapy clinic to initiate structured rehabilitation with a gradual weight-bearing program using an ankle-foot orthosis for four weeks. Initial exercises included ankle pumping and active ROM exercises, focusing on inversion and eversion. At this stage, the patient’s subtalar mobility showed marked improvement compared to the preoperative condition, and he was scheduled for muscle strengthening, functional retraining, and dynamic weight-bearing physical therapy at two months postoperatively. At four months postoperative, the patient returned to the outpatient clinic, where clinical assessment showed restoration of the medial longitudinal arch, along with successful achievement of active pronation and supination of the left foot, full joint ROM, and no pain during walking. The patient’s OxAFQ-C scores were 83.3% in the physical domain, 87.5% in the school and play domain, and 87.5% in the emotional domain.

## Discussion

In this case, the talocalcaneal coalition was of a mixed type, comprising a posterior extra-articular osseous bridge together with a fibrous connection along the medial aspect of the talar facet joint. While most talocalcaneal coalitions involve the middle facet and are either purely osseous or non-osseous, the coexistence of different tissue types in distinct regions of the same coalition is rarely reported [13]. This unusual anatomy had important surgical implications. The fibrous component was located on the medial side of the subtalar joint, extending from the sustentaculum tali to the medial tubercle of the talus. Anatomically, the coalition was positioned posterior to the flexor digitorum longus and anterior to the FHL, with the neurovascular bundle lying adjacent to the posteromedial aspect of the FHL. To ensure safe and effective resection while minimizing the risk of tendon or neurovascular injury, a posteromedial surgical approach was selected, unlike the medial approach that is commonly used for middle-facet coalitions and is often limited by difficulty in visualizing the coalition [14]. The posteromedial route provides direct access to both middle- and posterior-facet coalitions without obstruction, despite the complex anatomical positioning of the surrounding structures. Additionally, it may reduce the risk of tendon injury and neurovascular damage [15].

Compared with the classic medial approach to the middle facet, the posteromedial route provides more direct access to both the middle and posterior facets without excessive dissection of the neurovascular bundle. This facilitates complete visualization of mixed posterior and medial coalitions and reduces the risk of iatrogenic injury to the FHL and tibial neurovascular structures. Endoscopic or arthroscopic resection has also been described and offers the advantage of smaller incisions and potentially faster recovery. However, it requires advanced equipment, has a steep learning curve, and can limit the ability to manage extensive osseous bridging. In our case, the posteromedial open approach allowed safe and thorough excision of both bony and fibrous components, while permitting secure placement of the fat graft.

The size of the coalition plays a crucial role in deciding between coalition resection and arthrodesis. However, hindfoot stiffness must also be taken into account, as it can increase the likelihood of excision failure. In general, the older the patient, the greater the probability that the hindfoot is stiff and that adaptive changes, such as arthritis and morphological alterations of the hindfoot joints, have developed, leading to a reduction in the chances of a successful excision [16].

Resection of a talocalcaneal coalition is an effective, yet technically demanding and challenging procedure, requiring a comprehensive understanding of subtalar joint anatomy and precise intraoperative three-dimensional orientation [17]. Ossification of the talonavicular coalition typically begins between three and five years of age, the calcaneonavicular coalition between 8 and 12 years, and the talocalcaneal coalition between 12 and 16 years [18]. In this study, we performed talocalcaneal coalition resection in a nine-year-old patient due to failed conservative management, functional impairment, and inability to participate in sports. Furthermore, removing the tarsal coalition allows the subtalar joint to regain its motion and the hindfoot to realign, which subsequently helps the medial longitudinal arch rise again. Gu et al. described a series of 16 patients with talocalcaneal coalition accompanied by pes planus who underwent coalition excision combined with extra-osseous talotarsal stabilization. Their outcomes demonstrated clear improvement in medial arch height and radiographic indicators, such as Meary’s angle, reflecting restoration of the medial longitudinal arch. This correction is attributed to the removal of the rigid osseous or fibro-osseous union, which allows the calcaneus to invert and the talus to plantarflex normally, reducing hindfoot valgus and reestablishing the soft-tissue and ligamentous support essential for maintaining the arch [19].

In this report, we performed talocalcaneal coalition resection with fat interposition to address the associated pes planovalgus, resulting in favorable clinical outcomes. Fat and bone wax interposition have been shown to offer superior pain relief, improved functional outcomes, and lower recurrence rates. However, bone wax carries a potential risk of foreign body reaction and increased susceptibility to infection [20]. Regarding interpositional grafts used in talocalcaneal coalition resections, gluteal or abdominal fat is the most commonly utilized material. Other options include bone wax and pedicled flaps of the tibialis posterior tendon sheath [3]. Tendon interposition using a split of the FHL or tibialis posterior tendon can be employed to fill the gap and help prevent recurrence. However, this technique carries potential complications, including limited active motion of the foot or great toe, tendon rupture, and hallux deformity [21]. McCormack et al. reported nine symptomatic talocalcaneal coalition cases treated with complete resection and fat graft interposition, with an average follow-up of 11.5 years. Most patients had a full ROM and no radiographic signs of degeneration [22]. Similarly, Gantsoudes et al. reported on 49 feet that underwent resection of talocalcaneal coalition with fat graft interposition, with a minimum follow-up period of 12 months. Based on the American Orthopaedic Foot and Ankle Society (AOFAS) Ankle-Hindfoot score, 32 cases had excellent outcomes, 10 had good outcomes, 6 were rated as fair, and 1 had a poor outcome. When outcomes were further analyzed according to follow-up duration - one to two years, two to four years, and more than four years - there were no significant differences in AOFAS scores between the groups. Additionally, they concluded that coalition excision is the most effective approach to restore the ROM and alleviate pain [23]. Mosca reported that 87% of patients who underwent tarsal coalition resection achieved good outcomes [24]. A similar study conducted in Ontario, Canada, involving resection of calcaneonavicular and talocalcaneal coalitions, found that 85% of patients did not require any additional surgical procedures [25]. A recent systematic review reported a recurrence rate of 7% when fat was used for interposition [21]. A prospective study followed 16 feet in 15 children with talocalcaneal coalition treated arthroscopically, with a follow-up period of more than two years. The AOFAS score improved from 56.8 to 90.9, and no recurrences were observed. Furthermore, this approach offers the benefits of minimally invasive visualization while allowing direct evaluation of the subtalar joint. Compared with open surgery, it is associated with less postoperative pain, shorter hospital stay, lower infection and wound-related complication rates, and a faster overall recovery. However, it demands advanced hindfoot arthroscopy skills, and because the surgeon must operate in deeper, confined spaces, the risk of injury to nearby anatomical structures is increased. The posteromedial approach provides unobstructed access to both middle- and posterior-facet coalitions. Although the flexor digitorum longus lies anteriorly, the FHL lies posteriorly, and the neurovascular bundle sits just posteromedial to the coalition, adequate working space can still be created. This is attributed to the increased perfusion pressure in the region, which allows safe instrument manipulation directly at the coalition site and helps reduce the risk of tendon or neurovascular injury. In contrast, the medial approach typically uses an incision extending from the tip of the medial malleolus toward the sustentaculum tali. After exposing and resecting the coalition, the defect is usually filled with a fat graft or bone wax. Despite its familiarity, this method has notable drawbacks, including the potential for incisional neuroma, superficial wound infection, delayed wound healing, and the need for longer hospitalization to manage the wound and postoperative pain. The fat graft occupies the space created after the coalition is removed, serving as a barrier that helps prevent reossification [12,22].

Mixed-type talocalcaneal coalition in a single foot is an uncommon and often under-recognized condition. It can serve as a persistent source of foot pain in pediatric patients, making early recognition of its clinical pattern crucial for timely diagnosis [26,27]. Clinicians and radiologists should remain vigilant for this variant when evaluating suspected coalitions. Advanced imaging, particularly CT or MRI, is essential for accurately defining the coalition’s composition and extent, as well as for planning between resection and arthrodesis. Moreover, postoperative assessment should extend beyond subjective symptom improvement; incorporating objective functional measures and validated scoring systems offers a more reliable evaluation of treatment outcomes.

A key limitation of this report is the relatively short follow-up period of four months. Although the patient demonstrated excellent early functional recovery and restoration of subtalar motion, longer observation is required to fully assess the durability of the fat interposition, monitor for potential recurrence of the coalition, and identify any late degenerative changes. The short follow-up reflects that the case is still under active clinical surveillance. We plan to continue monitoring the patient and have scheduled further imaging, preferably CT or MRI, at approximately 12 months to evaluate graft integrity and long-term outcomes.

## Conclusions

Mixed-type coalition in the same foot is an uncommon and frequently under-recognized condition. Early diagnosis is essential to prevent progression, while advanced imaging is crucial for precise identification of the coalition and optimal surgical planning. In pediatric patients presenting with symptomatic mixed-type talocalcaneal coalition, surgical excision with fat graft interposition has proven effective in restoring joint mobility, relieving pain, and correcting deformity. Ongoing long-term follow-up is important to ensure continued pain relief, functional recovery, and proper alignment, ultimately leading to an improved quality of life for the child.
